# Immunohistochemistry for Diagnosis of Metastatic Carcinomas of Unknown Primary Site

**DOI:** 10.3390/cancers10040108

**Published:** 2018-04-05

**Authors:** Janick Selves, Elodie Long-Mira, Marie-Christine Mathieu, Philippe Rochaix, Marius Ilié

**Affiliations:** 1 Département D’anatomie et Cytologie Pathologiques, Institut Universitaire du Cancer-Oncopole, 1 Avenue Irène Joliot-Curie, 31059 Toulouse CEDEX 9, France; selves.j@chu-toulouse.fr (J.S.); Rochaix.Philippe@iuct-oncopole.fr (P.R.); 2 Laboratoire de Pathologie Clinique et Expérimentale, Hôpital Pasteur, FHU Oncoage, Université Côte d’Azur, 30 Voie Romaine, 06000 Nice, France; long-mira.e@chu-nice.fr; 3 Département de Biologie et Pathologie Médicales, Institut Gustave Roussy, 114, rue Edouard Vaillant, 94805 Villejuif, France; Marie-Christine.MATHIEU@gustaveroussy.fr

**Keywords:** carcinoma, unknown primary site, diagnosis, immunohistochemistry

## Abstract

Immunohistochemistry has become an essential ancillary examination for the identification and classification of carcinomas of unknown primary site (CUPs). Over the last decade, the diagnostic accuracy of organ- or tumour-specific immunomarkers and the clinical validation of effective immunohistochemical panels has improved significantly. When dealing with small sample sizes, diagnostic accuracy is crucial, particularly in the current era of targeted molecular and immune-based therapies. Effective systematic use of appropriate immunohistochemical panels enables accurate classification of most of the undifferentiated carcinomas as well as careful preservation of tissues for potential molecular or other ancillary tests. This review discusses the algorithmic approach to the diagnosis of CUPs using CK7 and CK20 staining patterns. It outlines the most frequently used tissue-specific antibodies, provides some pitfalls essential in avoiding potential diagnostic errors and discusses the complementary tools, such as molecular tumour profiling and mutation-specific antibodies, for the improvement of diagnosis and prediction of the treatment response.

## 1. Introduction

In the absence of an identifiable primary tumour site, despite extensive multidisciplinary investigations, carcinomas of unknown primary site (CUPs) are characterized as metastatic carcinomas [1]. Diagnosis is intended essentially to identify the subsets of CUPs sensitive to specific treatment. Beside these clinical entities, the identification of the primary tumour has no prognostic or therapeutic effect and a comprehensive systematic review is unnecessary and costly [2]. However, the therapeutic choice, and several favourable subsets of CUPs, warrants further histopathological characterization, which is often performed with immunohistochemistry (IHC) and, more recently, using molecular analyses [3,4]. In practice, four primary sites (breast, ovarian, prostate and thyroid) involving specific effective treatment options and a better prognosis should first be investigated [5]. In addition, the development of targeted therapies must eliminate a pulmonary or colorectal origin.

IHC provides diagnostic guidance in approximately 90% of undifferentiated malignant tumours but usually at the end of a fastidious and expensive algorithm based on both morphology and IHC. However, identification of the primary site of origin may represent a difficult challenge for the pathologist when dealing with a small sample size along with increased generation of tumour-specific primary antibodies and the need for complementary molecular analysis.

If morphology is not sufficient, in particular for an undifferentiated neoplasm with no clear lineage differentiation, the first diagnostic IHC panel will include a few antibodies directed against epithelial antigens (broad spectrum anti pan-cytokeratin antibody cocktail, for example, AE1/AE3, KL1, pan-Cytokeratin Plus [AE1/AE3+ 8/18]), lymphoid antigens (CD45 or CD20 and CD3) and melanocyte-differentiation antigens (S100 protein, SOX10) [6]. Vimentin is a nonspecific marker; however, a vimentin-negative tumour is unlikely to be a sarcoma (with the exception of alveolar soft part sarcoma), lymphoma, or melanoma [7].

Among the carcinoma metastases, IHC will distinguish three histopathological subtypes: (i) adenocarcinoma (≈50% of cases), (ii) poorly differentiated carcinoma (≈30% of cases) and (iii) squamous cell carcinoma (≈15% of cases). As coordinate expression of CK7 and CK20 defines subsets of carcinomas, the next step will be to determine the likely primary site based on their expression profiles (Table 1) [4,8].

However, the expression of cytokeratin is not restricted to epithelial neoplasms [9]. Keratin is commonly expressed in some tumours with evidence of epithelial differentiation such as synovial sarcomas, epithelioid sarcomas, desmoplastic small round cell tumours, chordomas, adamantinoma and myoepithelial carcinomas. In addition, aberrant focal expression of cytokeratin has been reported in other tumours including angiosarcomas, epithelioid hemangioendotheliomas, epithelioid leiomyosarcomas and meningiomas, undifferentiated pleomorphic sarcomas, rhabdomyosarcomas, malignant rhabdoid tumours and peripheral nerve sheath tumours, clear cell sarcomas, plasmacytomas, diffuse large B-cell lymphomas, anaplastic large cell lymphomas and melanomas [9].

On the other hand, some tumour-specific antibodies may show a less intense signal or no signal at metastatic sites. The poorly differentiated malignant phenotype can vary but rare positivity is generally weak and focal. In addition, the intra-tumour heterogeneity can make interpretation more difficult. It is for this reason that IHC using a more or less extensive panel and specificity of antibodies for certain tumour types is necessary in order to precisely determine the origin. In addition to CK7 and CK20 cytokeratins, some of the markers often used routinely are more specific for the primary site.

No single antibody is fully sensitive and specific for a particular tumour; however, some antibodies are especially useful when used within small panels [7]. Over the past few years, novel antibodies have been developed, essentially those directed against transcription factors and improved the specificity of diagnosis, when used in combination with other more “classical” antibodies [7,10]. However, in approximately 5% of cases, IHC may not provide any definitive information and the final diagnosis of an undifferentiated malignant neoplasm is given without any reference to the tumour origin, despite all the investigations.

The amount of relevant information required by the clinician to provide optimal and personalized therapeutic support is increasing and transcends the question of the origin of the primary tumour [1]. As of now, platinum-based chemotherapy is the standard management for patients with CUPs. More recently, an alternative strategy offers treatment targeting the assumed primary tumour according to its molecular signature [11]. With this in mind, the randomized phase III trial GEFCAPI04 comparing standard chemotherapy to a chemotherapy regimen tailored to the primary site defined by the gene expression profile is currently recruiting patients in France (clinicalTrials.gov NCT01540058) [1].

Overall, the final diagnosis of CUPs is performed on small-sized samples that are essential to ensure that complementary IHC tests and molecular analyses can be performed without prejudice to the management of the patient. In addition, the myriad of IHC markers required to guide the diagnosis may question their availability and the budgetary impact for each pathology laboratory as well as the potential implementation at a national level of ‘IHC expert centres’ to provide second opinion IHC services.

## 2. Diagnostic Workflow of CK7+/CK20− CUPs

For CUPs with a CK7+/CK20− pattern, a gastrointestinal origin is diagnosed in 2/3 of cases (50% colorectal, 20% pancreatic, 10% gallbladder, small intestine and rectum). In the 1/3 of remaining cases, more than 50% had an ovarian origin, followed by a lung or breast origin [12]. In only a few cases, it may be difficult to distinguish the primary origin of tumours including mesothelioma [12].

Diagnosis is based on knowledge of the clinical record of the patient, including the clinical oncological history but also the age and sex. For instance, the search for the expression of hormone receptors is essential for a female patient but is has limited interest for a male patient. Likewise, the expression of WT1 in a female patient can guide the diagnosis either to an ovarian serous carcinoma or malignant mesothelioma; whereas WT1 expression in a male will strongly favour a mesothelioma. Finally, a tumour in an adolescent or young adult is in favour of germ cell, lymphoid, desmoplastic small round cell tumour or Ewing’s sarcoma rather than an ovarian epithelial tumour, for example.

The staining pattern for CK7 and CK20 gives an overall indication but has some limitations. For instance, gastric carcinomas can present a highly variable profile: CK7+/CK20+ in 32% of cases; CK7−/CK20+ in 35% of cases and CK7−/CK20− in 14% of cases [8]. Likewise, ductal and lobular breast carcinomas have a CK7+/CK20− pattern in 86% to 94% of cases but other patterns have been described in a decreasing order of frequency, for example CK7+/CK20+, CK7−/CK20+ and CK7−/CK20− [8].

Once the CK7/CK20 expression profile is established, complementary organ-specific antibodies allow refinement or more precise guidance toward the primary origin of CUPs. However, their reactivity is often complex and they should be used as an integral part of a panel (Table 2) [7]. The most classical antibodies with complex reactivity, used when dealing with a CUP CK7+/CK20− are: TTF1, GATA3, PAX8 and WT1 [13].

**Figure 1:**
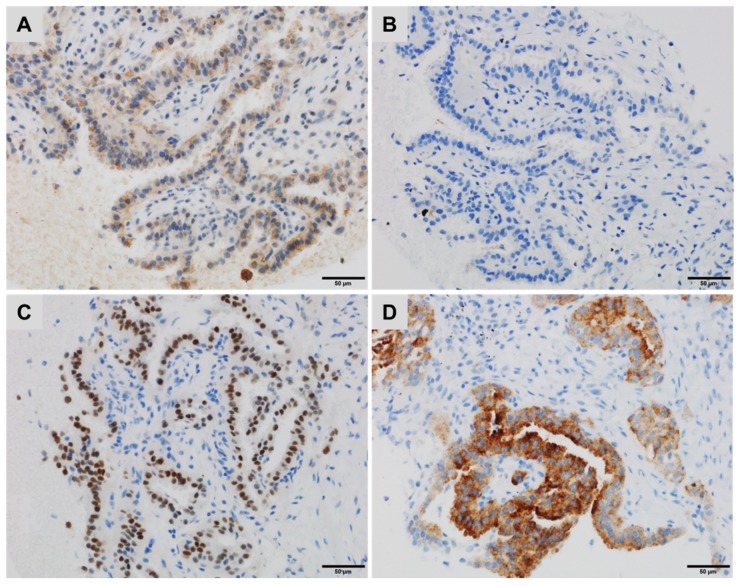
A lung metastasis of a thyroid carcinoma with a micropapillary component showing (**A**) cytoplasmic staining for Napsin A, (**B**) negative staining for Thyroglobulin, (**C**) diffuse, strong nuclear staining for PAX8 and (**D**) strong cytoplasmic staining for BRAFV600E (immunoperoxidase, clone VE1).

TTF1 is a nuclear transcription factor that promotes embryogenic pulmonary and thyroid differentiation and is expressed by most but not all, lung or thyroid neoplasms [22]. TTF1 expression decreases in poorly differentiated (~50%) compared with well-differentiated (~100%) adenocarcinomas, diminishing the sensitivity of this marker to identify the pulmonary origin of a poorly differentiated adenocarcinoma [23]. Among the thyroid carcinomas, most of the undifferentiated anaplastic ones are not immunoreactive for TTF1 (positive in less than 5%) [24]. In addition, a minority of anaplastic and poorly differentiated thyroid carcinomas may express Napsin A, which is more common in thyroid carcinomas with a micropapillary component, a pattern shared with some lung adenocarcinomas. PAX8 may be diagnostically useful to distinguish these Napsin A-positive and Thyoglobulin-negative thyroid carcinomas from lung adenocarcinomas, which are PAX8 negative (Figure 1) [24,25].

**Figure 2:**
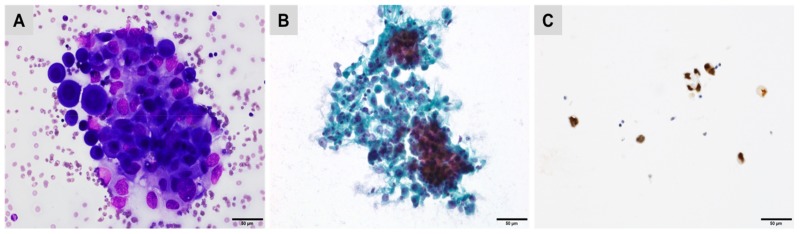
Pleural effusion showing reactive mesothelial cells mixed with metastatic urothelial carcinoma cells ((**A**) Giemsa; (**B**) Papanicolaou staining) demonstrating strong nuclear staining for GATA3 (**C**).

GATA3 was suggested to identify a breast origin in a tumour expressing CK7+/CK20− and hormone receptors. GATA3 is expressed in 92% to 100% of ductal and lobular breast carcinomas [14,15]. It exhibits nuclear staining, with most often diffuse and strong reactivity. This staining pattern is observed regardless of the histological subtype (ductal or lobular), the type of sample (biopsy versus surgical resection sample) and the biological phenotype of the tumour (hormone receptor positive or negative and triple negative tumour phenotype) [16]. However, GATA3 is also expressed in normal urothelial epithelia and in 80% to 90% of urothelial carcinomas (Figure 2) [16].

In addition, other epithelial neoplasms commonly express GATA3 such as cutaneous basal cell carcinomas (98%), cutaneous squamous cell carcinomas (81%), cervical squamous cell carcinomas (33%), skin adnexal tumours (100%), mesothelioma (58%) and chromophobe renal cell carcinoma (51%) [17].

PAX8 is a nuclear marker, usually expressed in Müllerian tumours (ovarian and endometrial), renal cell carcinomas and thyroid carcinomas of follicular cell origin [18]. PAX8 is a useful marker to distinguish gynaecologic carcinomas from non-gynaecologic malignancies including malignant mesotheliomas, cancers of gastrointestinal origin and breast carcinomas CK7+/CK20− and hormone receptor-positive [19]. The PAX8 expression profile has been evaluated in two series of 1100 and 1200 normal and tumour tissues and demonstrated a strong sensitivity in ovarian carcinomas (79% to 99%), endometrial carcinomas (84% to 93%), cervical adenocarcinomas (84%), renal cell carcinomas (90% to 100%), thyroid carcinomas (100% of follicular, papillary and poorly differentiated, 79% of anaplastic carcinomas and 75% of medullary carcinomas) [20,21]. PAX8 was negative in 98% of cervical squamous cell carcinomas and in 93% of bladder carcinomas, including adenocarcinomas, while no expression was detected in adenocarcinomas of the breast, colon, prostate, liver, testicular, stomach, oesophagus, melanoma, gastrointestinal stromal tumours, leiomyosarcoma or pheochromocytoma [20,21]. Overall, PAX8 is a specific and sensitive marker for Müllerian tumours, renal cell, ovarian and thyroid carcinomas.

WT1 is a nuclear marker expressed in a heterogeneous tumour group such as malignant mesothelioma, serous ovarian carcinoma or round cell desmoplastic tumour [34]. Several studies have shown that WT1 is expressed in more than 90% of high-grade serous carcinomas of the ovary and more rarely in serous carcinomas of the endometrium. However, it can also be expressed in benign mesothelial tumours and malignant mesotheliomas. In the case of diagnostic doubt between a serous carcinoma or a mesothelioma, it will not be useful in women but can be valuable in men. Focal expression has been described in a minority of cases of gastric, pulmonary or urothelial carcinomas [34]. Special attention should be paid to the expression of WT1 in breast carcinomas but these cases demonstrate a cytoplasmic and not a nuclear reactivity [34].

Moreover, the first diagnosis to evoke when dealing with peritoneal carcinomatosis CK7+/CK20− is that of a malignant peritoneal mesothelioma (MPM) and in such cases IHC is an essential diagnostic tool [33]. The choice of antibodies will depend on the histological type (epithelioid versus sarcomatoid), the topography (pleural versus peritoneal) and the expected differential diagnosis. The differential diagnoses to be considered for epithelioid MPMs are essentially peritoneal carcinomatosis from a primary serous carcinoma of the peritoneum/ovary/Fallopian tubes, gastrointestinal tract and less frequently of pulmonary, mammary or uterine origin. All epithelioid MPMs express pan-cytokeratin (e.g., AE1/AE3, KL1, MNF16). Thus, if a malignant peritoneal neoplasm of epithelioid morphology does not express cytokeratin, other diagnoses should be considered. It is necessary to use a panel of so-called “positive” and “negative” MPM antibodies (Table 3) [31,32].

Thus, the positive markers for MPM are: Calretinin (with the “fried-egg” appearance or cytoplasmic staining with nuclear reinforcement), CK5/6 (cytoplasmic staining), EMA (membranous staining), WT1 (nuclear staining) and D2-40 (membranous staining). The negative markers for MPM or “epithelial markers” are: BerP4, B72.3, MOC-31, BG8, CEA, PAX8 (Müllerian carcinomas in women), hormonal receptors (in women) and prostate-specific antigen (PSA) in men.

The expression topography must be taken into account. For example, EMA has traditionally a membranous reactivity in MPMs while being localized in the cytoplasm in ADCs. There is no well-defined threshold of positivity in terms of percentage of marked cells but some authors have retained a minimum value of 10%.

The useful markers in women for MPMs are Calretinin and probably D2-40 (which may be positive in some cases of serous carcinoma) and MOC-31, BG8 (less specific) and BerEP4 in favour of ADCs. In men, the useful markers for MPM are Calretinin, WT1 (nuclear reactivity) and D2-40. Finally, the useful markers for a non-serous carcinoma are: B72.3, MOC31, BG8 and Ber-EP4.

The international expert group, the “International Mesothelioma Interest Group” recommends that two so-called “positive” markers and two “negative” markers be used as a first step to diagnosis, depending on the expected differential diagnoses [31,32]. If the results are consistent, a diagnosis can be made. In the event of a discrepancy, a second broader panel of antibodies is then required.

**Figure 3:**
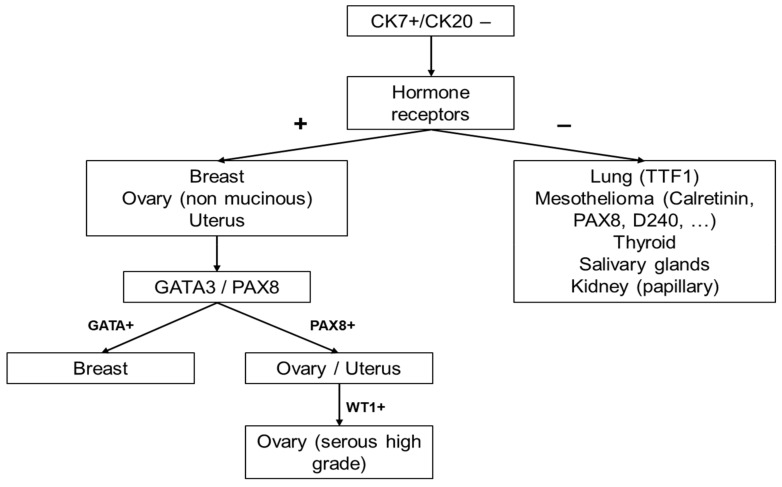
The diagnostic algorithm in female patients with CK7+/CK20− CUPs.

In summary, when dealing with a CK7+/CK20− CUPs without precise or reliable clinical information and depending on the morphological aspects, the CK7/CK20 profile will roughly orientate towards a primitive origin of the neoplasm. More organ-specific antibodies will be added such as ER, PR, PAX8 (Müllerian tumours), TTF1 (lung, thyroid), GATA3 (breast, urothelial), CDX2 (gastrointestinal) and Calretinin, BerEP4, WT1 and CK5/6 (malignant mesothelioma) (Figure 3) [10].

## 3. Diagnostic Workflow of CK7−/CK20+ CUPs

**Figure 4:**
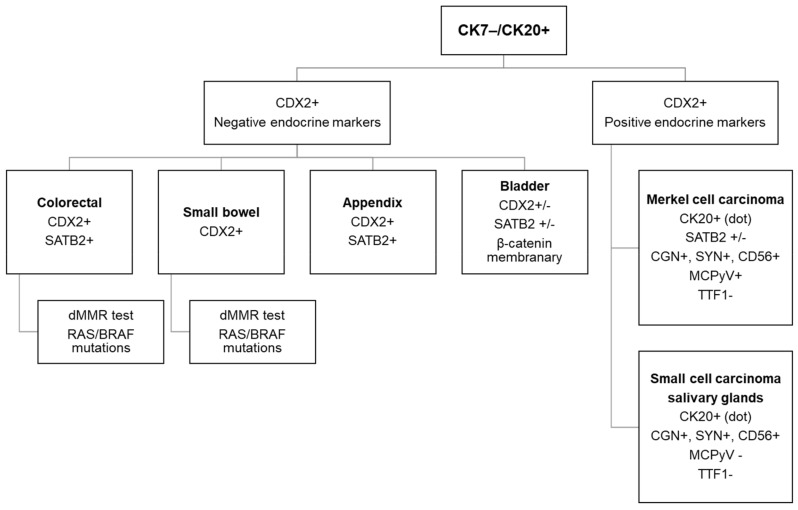
The diagnostic algorithm for workup of a CK7−/CK20+ carcinoma.

When dealing with a CK7−/CK20+ CUPs of non-Lieberkhunian morphology, the first aetiology to be considered is a colorectal origin corresponding most often to a poorly differentiated adenocarcinoma (Figure 4) [7].

In these undifferentiated forms, CK20 is often only focally positive, whereas medullary or undifferentiated carcinomas of colorectal origin frequently have a CK7−/CK20− phenotype. In the context of a CK7−/CK20+ CUPs, CDX2 has limited diagnostic value [26]. Actually, CDX2 is not an anatomical marker of the intestine but a transcription factor promoting differentiation and the expression of intestinal specific genes in intestinal cells. It is therefore a low-specific marker for a colorectal origin as it can be expressed in all carcinomas with intestinal differentiation such as adenocarcinoma of the small intestine but also in certain gastric, oesophageal, pancreatic, ovarian, urachal and sinonasal carcinomas [27]. CDX2 expression may be decreased in right-sided or proximally located colon carcinomas, in poorly differentiated colorectal carcinomas as well as in colorectal carcinomas with microsatellite instability-high (MSI-H) or mismatch repair deficient (dMMR) [28].

Differential expression of mucins (MUC2, MUC5AC, MUC6, MUC4) that distinguishes the different intestinal origins has been proposed by some authors [29]. However, the frequent association of the expression of several mucins in the same tumour, the difficulty in determining an expression threshold for each of them, as well as the absence of therapeutic impact of the identification of these anatomical origins limits their diagnostic interest [29]. Villin has no added value compared to CDX2 and can also be expressed in numerous non-intestinal carcinomas (bladder, Merkel cell carcinoma, sinonasal intestinal-type adenocarcinoma). On the other hand, the addition of a new marker, the transcription factor SATB2 (special AT-rich sequence-binding protein 2), seems more useful because it would be restricted to the lower gastrointestinal tract [29]. Its expression is maintained in almost all adenocarcinomas of the appendix (including adenocarcinoma ex-goblet cell carcinoid) and in over 85% of colon adenocarcinomas [29]. This increases the predictive value of a colon origin from 93% to 99% for CK7−/CK20+ tumours and is expressed in poorly differentiated colon tumours CDX2− and/or CK20− [35]. Moreover, its expression is preserved in the majority of colorectal medullary carcinomas [36].

It is often difficult but important to distinguish between an adenocarcinoma of the bladder from a colorectal origin because these two tumours have the same morphological appearance and immunohistochemical profile (CK7−/CK20+/CDX2+), while the therapeutic management is different. The reference treatment for bladder adenocarcinomas is surgical, as these tumours are poorly chemosensitive, unlike colon carcinomas [37]. In addition, the diagnosis of the urachal origin is based on clinical and morphological criteria, while the immunohistochemical profile is most often variable with low specificity, showing similarities to that of extravesical adenocarcinomas. The CK7/CK20 pattern is not very informative in this indication [38,39]. The CK7+/CK20+ phenotype is observed in approximately 50% of urachal adenocarcinomas, while almost 30% have no CK7 staining and a CK7−/CK20− phenotype can also be observed exceptionally [38,39,40,41]. A difference in activation of the Wnt/β-catenin pathway may be useful, resulting in nuclear staining for β-catenin in colorectal carcinomas (75% of cases), while it is mainly expressed in the cell membrane and cytoplasm in bladder adenocarcinomas (17%) [42].

**Figure 5:**
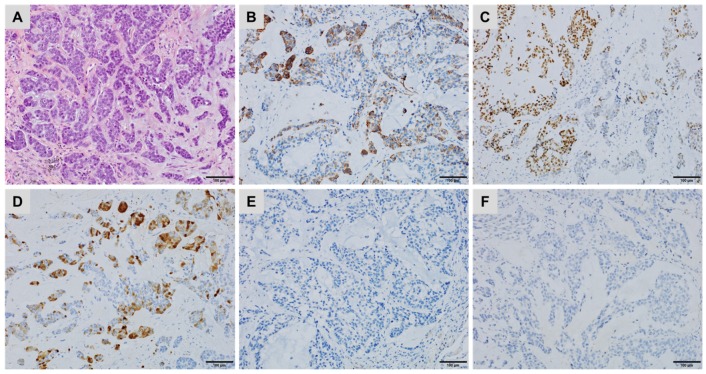
A metastatic urachal adenocarcinoma in the left lung ((**A**) haematoxylin, eosin and safran) showing patchy staining for (**B**) CK34βE12, (**C**) CDX2 and (**D**) Calretinin but no staining for (**E**) p40 and (**F**) GATA3 ((**B**–**F**) immunoperoxidase).

Thrombomodulin is expressed in most urothelial carcinomas and in approximately 60% of bladder adenocarcinomas of urachal or non-urachal type. Although positive in numerous other carcinomas, thrombomodulin is negative in digestive adenocarcinomas. CK34βE12 is expressed in approximately 60% of urachal adenocarcinomas and in only 10% of colorectal adenocarcinomas (Figure 5). Loss of membrane localization and aberrant nuclear E-cadherin expression is often associated with aggressive progression of certain carcinomas, including primary urachal adenocarcinoma [43]. Because it is often lost, or very focally expressed, p63 or p40 are unreliable markers for urachal adenocarcinomas (Figure 5) [39,44].

GATA3 is a marker of urothelial differentiation, expressed in 70% to 90% of urothelial carcinomas but is often lost in primary bladder adenocarcinomas (Figure 5), with the exception of signet-ring cell carcinoma of the urinary bladder. It is also expressed in a number of extravesical adenocarcinomas, including breast carcinomas [39,44]. SATB2 is expressed in almost half of the bladder adenocarcinomas, which limits the interest of this antibody in distinguishing between the two origins [29].

The immunohistochemical profile of a CK7−/CK20+ CUPs should include endocrine markers (Chromogranin, Synaptophysin and CD56) as this profile is unique to Merkel cell carcinomas and small cell carcinomas of the salivary glands, both with perinuclear “dot-like” CK20 staining. Therefore, the phenotype of Merkel cell carcinomas is CK7−/CK20+ (“dot-like”), CDX2−, SATB2+, Chromogranin+, Synaptophysin+ and positive for Merkel cell polyomavirus (MCPyV), while the phenotype of small cell carcinomas of the salivary glands is Synaptophin+, Chromogranin+/−, CD56+ and CDX2− [29,45].

MSI-H/dMMR colorectal carcinomas are characterized morphologically by poor differentiation or mucinous secretion and prominent lymphoid stroma but these characteristics are not constant. On the other hand, the morphology of medullary carcinoma is pathognomonic of a dMMR phenotype. The immunohistochemical profile of dMMR colorectal carcinomas can be variable: CK7−/CK20+ but also frequently CK7−/CK20− [46]. They are negative for CDX2 in 15% to 20% of cases [29]. SATB2 appears to be a more sensitive marker than CDX2 but its expression in the dMMR tumour group is not really known outside medullary carcinomas [36]. Only 5% of metastatic forms of colorectal cancers have a MSI-H/dMMR phenotype. This oncogenic pathway results from the loss of function of the dMMR genes, most often of sporadic origin (80% of cases) by somatic inactivation of the MLH1 gene (hypermethylation of the MLH1 gene promoter), more rarely with genetic harbouring of a deleterious germline mutation in one of the four MMR genes associated with the Lynch syndrome [47].

*BRAF* gene mutations in MSI-H colorectal cancers are associated with sporadic forms and can be used to rule out the Lynch syndrome. It should be pointed out that the MSI oncogenic pathway and the dMMR phenotype are not specific for a colorectal origin but are also associated with other types of carcinomas, primarily the endometrium but also gastric, upper urinary tract, small intestine and ovary cancers [47].

It is important to identify the colorectal origin of a metastatic neoplasm to propose the most appropriate therapy and to initiate molecular testing guiding this treatment: *RAS* mutations (*KRAS* and *NRAS* mutations on exons 2, 3 and 4) for the use of *EGFR* inhibitors, *BRAF* mutations on exon 15 for therapeutic intensification and dMMR testing for immunotherapy. Currently, the treatment of metastatic colorectal carcinoma corresponds to bi-chemotherapy (5FU and oxaliplatin) combined with targeted therapy (anti-angiogenic or anti-*EGFR*) or tri-therapy (5FU, oxaliplatin, irinotecan +/− anti-angiogenic therapy) for aggressive forms, which is guided by several biomarkers. Recent data of clinical trials suggest that the dMMR status should be performed upon diagnosis of metastatic colorectal carcinoma for treatment with anti-PD-1 immunotherapy [48]. While known to have a good prognosis in non-metastatic forms, dMMR colorectal carcinomas have poor prognosis at a metastatic stage and patients must benefit promptly from immunotherapy [48].

In summary, the potential origins of CK7−/CK20+ CUPs are limited: most often colorectal (or digestive), non-digestive intestinal (bladder), or endocrine (Merkel cell carcinoma and small cell carcinoma of salivary glands), requiring a limited antibody panel to distinguish them: CDX2 (+/− SATB2), endocrine markers and MCPyV. MSI-H colon cancers are often poorly differentiated, have lost the markers of intestinal differentiation (e.g., CK20, CDX2) but have an aggressive behaviour at a metastatic stage, possibly benefiting from immunotherapy. Identifying the colorectal origin of a metastatic carcinoma is rarely difficult, except in poorly differentiated cancers but extremely useful for optimal treatment.

## 4. Diagnostic Workflow of CK7+/CK20+ CUPs

In the event of malignant epithelial proliferation with a diffuse or focal CK7+/CK20+ profile, the pulmonary origin (mucinous adenocarcinoma ex-bronchioalveolar carcinoma and pulmonary intestinal-type adenocarcinoma), pancreatic, digestive (gastric, oesophageal, small intestine), ovarian (mucinous sub-type) and sometimes bladder or midline origin should be excluded (Table 4) [1,7].

Pulmonary metastases have a prevalence of 30% to 50% in patients with extra-thoracic neoplasms. Clinically, the most frequent primitive origins are breast, colon, gastric, pancreatic, renal, melanocytic, prostate, prostate, liver, thyroid, adrenal and genital. The CK7+/CK20+ pattern allows the exclusion of breast (CK7+/CK20−), colon (CK7−/CK20+/−), renal (CK7−/CK20−), prostate (CK7+/CK20−), hepatic (CK7−/CK20−), thyroid (CK7+/CK20−) and adrenal (CK7−/CK20−) origins [1,7].

**Figure 6:**
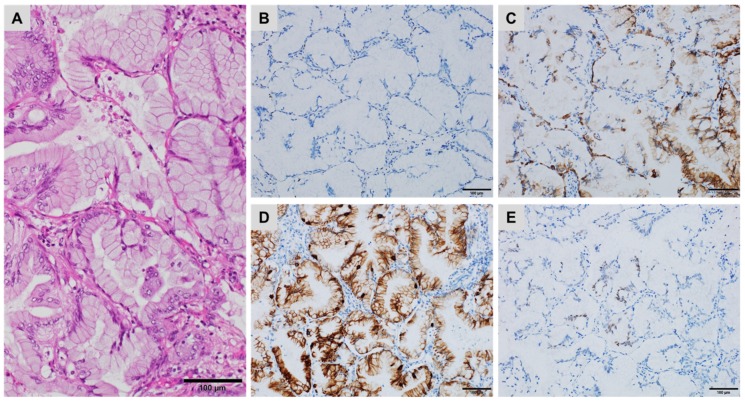
Primary mucinous pulmonary adenocarcinoma ((**A**) haematoxylin, eosin and safran) showing (**B**) no staining for TTF1, (**C**) patchy staining for CK7, (**D**) strong staining for CK20 and (**E**) a few tumour cells stained for CDX2 ((**B**–**E**) immunoperoxidase).

Mucinous pulmonary adenocarcinomas usually develop in the peripheral regions of the lungs. Several patterns have been described such as lepidic, acinar, papillary, micropapillary, solid and cribriform like in other adenocarcinomas [49]. In general, they are TTF1 negative and can lose the expression of CK7, associated with expression of CK20 and sometimes CDX2 (Figure 6).

A *KRAS* mutation was found in 56% of mucinous adenocarcinomas, while the mutational status correlated neither with the architectural pattern nor with survival of patients [49]. The main differential diagnosis is the metastasis of pancreatic or ovarian origin, with clinical information being essential [49].

The pulmonary intestinal-type of adenocarcinoma is less frequent. The architecture is cribriform or acinar with tubulo-papillary aspects and may present with focal necrotic points. The cells are usually cylindrical with a brush border and elongated and pleomorphic hyperchromatic nuclei and nuclear crowding. This tumour often mimics a conventional colorectal adenocarcinoma, which is the main differential diagnosis [49]. IHC is not very contributory in this histological TTF1− subtype. Interestingly, TTF1 are rarely expressed in genuine colorectal adenocarcinoma, therefore the clinical information remains paramount. 

NUT midline carcinomas are very aggressive tumours harbouring rearrangements in the *NUT* gene that can be detected by IHC using an anti-NUT-specific monoclonal antibody [53,54]. Although initially described in young subjects, this neoplasm can affect all ages (2–78 years) without gender predominance. This carcinoma does not present any tissue or organ specificity and is morphologically indistinguishable from other poorly differentiated carcinomas originating from midline locations (e.g., epiglottis, sinonasal, lung, mediastinum). The tumours have abrupt areas of squamous differentiation and frequently expresses the p40 protein [53]. NUT midline carcinomas comprise a group of highly aggressive tumours and they are accompanied by distant metastases at the time of diagnosis [55].

The IHC pattern for CK7+, CK20+, GATA3+, p40+ (inconsistent), Villin−, Thrombomodulin+ and the cytoplasmic expression of β-catenin point to a bladder/urothelial origin. Conversely, a Villin+ and Thrombomodulin+ profile combined with nuclear positivity for β-catenin and membrane reactivity to CDH17 are in favour of an adenocarcinoma of the small intestine [39].

The expression of CEA, CA. 125, Dpc4 (SMAD4 family, tumour suppressor gene) or MUC2 but not MUC5AC, would argue in favour of an ovarian metastatic origin. Moreover, WT1, which are expressed by mesothelial, ovarian (granulosa) and renal glomerular cells, are of interest for CUPs based on the high sensitivity and specificity (>90%) in serous carcinomas of the ovary [52]. In addition, Dpc4 is also expressed by normal pancreatic tissue (ducts, acinar cells). A loss of expression is in favour of malignant transformation, which is highly useful for biopsies. However, while not very specific, this marker has little interest for CUPs as it can also be expressed in the metastases of colorectal, appendix, gastric and endocervical carcinomas [7].

A panel of four markers, associating positivity for Maspin A (mammary serine protease inhibitor), S100P (placental S100 protein), IMP-3 (insulin-like growth factor II messenger RNA binding protein-3) and negativity for pVHL (von Hippel-Lindau tumour suppressor), has 100% sensitivity and specificity for distinguishing high grade dysplasia or malignancy from reactive atypia within the biliary-pancreatic system [50]. In addition, pVHL has added value to distinguish intrahepatic biliary malignancy (cholangiocarcinoma, pVHL positive) from extrahepatic biliary or pancreatic metastasis (pVHL negative). However, the individual expression of each of these markers does not provide a diagnostic element. For instance, the specificity of S100P is limited as many neoplasms demonstrate positive expression, including pancreatic, gallbladder, digestive, bladder and pulmonary adenocarcinomas [51]. In addition, their routine use is limited by their restricted availability in pathology laboratories. Finally, activating mutations in the *KRAS* oncogene are likely the single most common genetic abnormality in pancreatic cancer, present in ~90–95% of cases. Despite the low specificity, a *KRAS* gene mutation could provide additional diagnostic criteria [56].

In summary, a diffuse or focal CK7+/CK20+ profile favours a pulmonary (mucinous or intestinal subtypes), pancreatic, gastrointestinal (gastric, oesophageal, small intestine), ovarian (mucinous subtype), bladder or midline carcinoma origin. The absence of a specific marker or the scarcity of certain markers makes clinical information essential, in particular to differentiate a primitive pulmonary origin from a secondary digestive or pancreatic origin.

## 5. Diagnostic Workflow of CK7−/CK20− CUPs

When confronted with an adenocarcinoma morphology, the CK7−/CK20− profile excludes a primary pulmonary origin, while prostate, renal, liver or adrenal origins may be proposed (Table 5) [57].

**Figure 7:**
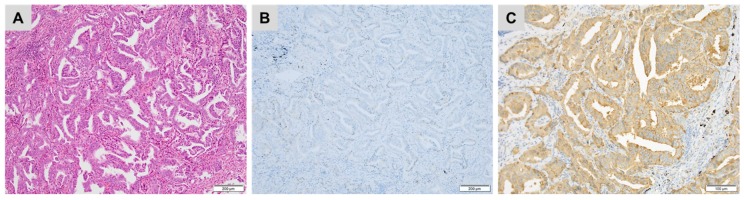
A metastatic prostatic adenocarcinoma (**A**) haematoxylin, eosin and safran) showing (**B**) no staining for prostate-specific antigen (PSA) but (**C**) diffuse, strong cytoplasmic staining for prostatic specific acid phosphatase (PSAP) ((**B**,**C**) immunoperoxidase).

In the absence of clinical information, a prostatic origin may be suspected based on the morphology in the case of well-limited tumour proliferation, arranged in small tubular structures composed of medium-sized uniform cells with prominent nucleoli. Necrosis, mitosis and vascular invasion are usually rare or absent. The use of anti-PSA and anti-PSAP (prostate-specific acid phosphatase) antibodies allows confirmation of a prostatic origin in most cases [58]. PSA is expressed in normal prostate tissue and in most prostatic adenocarcinomas [58]. However, in high-grade or poorly differentiated carcinomas and in certain histological subtypes such as small cell carcinoma these markers may be negative. In addition, the loss of PSA expression can be observed in 10% to 20% of metastases and after hormone therapy (Figure 7). Finally, PSA expression has been described in other types of carcinomas such as salivary gland or breast carcinomas [59]. PSAP may be a useful additional marker (Figure 7). It is also expressed in normal prostate tissue and in a majority of prostatic adenocarcinomas. However, it has a lower specificity than PSA, due to its expression in neuroendocrine tumours (pancreas, digestive carcinoid tumour), urothelial or cloacogenic carcinomas [60].

Other markers, described as highly specific of a prostate origin, can also be used alone or in combination to determine a prostate origin, such as P501S (prostein), PSAM (prostate-specific antigen membrane) and NKX3.1 [62]. Recently, the detection of the ERG protein, which is related to the presence of the fusion gene TMPRSS2-ERG, the most common and specific molecular alteration in prostate cancer, appears to be a complementary diagnostic argument in CUPs, since it is detected in approximately 30% of prostate metastases [61]. Finally, the androgen receptor has a low specificity, as it is expressed in several other tumour types (e.g., breast carcinoma, sebaceous carcinoma) and therefore cannot be used alone to confirm the diagnosis [30].

**Figure 8:**
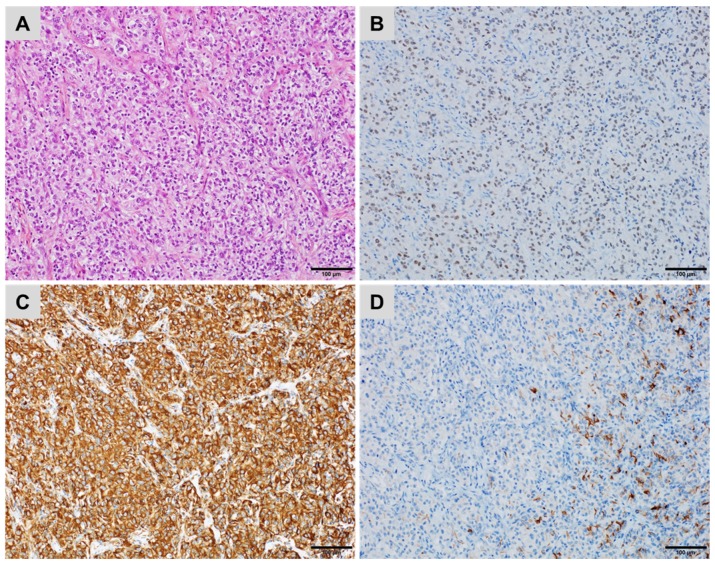
A metastatic renal cell carcinoma ((**A**) haematoxylin, eosin and safran) showing (**B**) diffuse nuclear staining for PAX8, (**C**) diffuse, strong cytoplasmic staining for Vimentin but (**D**) only a few tumour cells stained for CD10 ((**B**–**D**) immunoperoxidase).

The CK7−/CK20− profile should also eliminate metastasis from a clear cell renal cell carcinoma. Pulmonary metastases of renal origin are often poorly differentiated or may have different morphological characteristics compared to the primary tumour. A panel of several antibodies associating CK7 and CK20, Vimentin, the RCC marker, CD10 and CAIX most often confirm a renal origin (Figure 8) [63]. PAX8 is a sensitive but not very specific marker, its expression has been observed in carcinomas of renal, thyroid, ovarian, endometrial, or uterine origin (Figure 8) [21].

Finally, useful markers in favour of a hepatocellular carcinoma (HCC) are HepPar-1 and the canalicular markers CD10 or polyclonal CEA. They are expressed in the majority of HCCs (>80%) but sometimes in a heterogeneous way and mostly in well and moderately differentiated HCCs [64]. Arginase-1 is more sensitive (>90% of HCCs) and has the advantage of being reactive in poorly differentiated HCCs but may be less available in pathology laboratories. AFP and Glypican-3 are less sensitive but somewhat more specific oncofetal markers (Glypican-3 can be expressed in some melanomas). However, they have the advantage of being expressed in some poorly differentiated HCCs that do not express HepPar-1 or canalicular markers. HCCs express some cytokeratins (CK8, CK18) but pan-cytokeratins are often negative and they do not generally express EMA [64].

## 6. Complementary Tools for Diagnosis and Prediction of Treatment Response in CUPs

Despite advances in imaging and IHC, CUPs still account for approximately 3% of adult cancers; they are aggressive (medium-average survival <12 months) and heterogeneous forms of cancer. The CK7+/CK20− phenotype, the most frequent (~60%) adenocarcinomas of unknown origin, has been chosen to introduce new molecular biology techniques that can complement IHC in cases of undifferentiated or non-specific forms of CUPs [65,66]. In theory, the molecular analysis orientates towards a primary origin and to specific treatment strategies, adapted to the primary cancer, which could then be proposed to the patient [11,65,66].

Based on their site of origin specific gene expression profiles are now recognized in cancers, reflecting the different gene expression profiles present in the normal tissues of origin [67,68]. Several gene signatures using reverse transcriptase polymerase chain reaction (RT-PCR) or a microarray-based gene expression analysis have been proposed [69,70]. These assays were validated on tumour series of known primary origin and now allow determination of sites of origin for CUPs by analysing the rate of concordance of the genomic profile with that of a metastasis of known origin.

A 92-gene real-time RT-PCR assay (CancerTYPE ID^®^; bioTheranostics, Inc., San Diego, CA, USA) can identify the primary cancer for metastases of known origin in 85% of cases and for CUPs for which the primary origin was found in 15/20 cases (75%) [70,71]. In a prospective series of 247 CUPs, 98% of cases had a tissue of origin predicted by the assay [11].

However, the technique has a failure rate of 13% due to insufficient tumour material. The most frequently identified primary sites (~55%) are biliary, urothelial, colorectal and pulmonary. The primary origin is identified with a probability superior to 80% in 48% of cases. When the test predicts a more treatment-sensitive type of cancer, the median survival is improved significantly compared to other tumour types (13.4 versus 7.6 months, respectively; *p* = 0.04) [11].

However, this prospective trial did not answer the question as to whether a treatment adapted to the primary tumour identified by molecular analysis influences the outcome of patients. The currently ongoing European randomized, phase III, multi-centric trial GEFCAPI04 aims to answer this question (Clinicaltrials.gov/NCT01540058) [72,73]. This study compared a diagnostic and therapeutic strategy based on molecular analysis followed by tailored specific therapy for a suspected primary cancer, to an empiric strategy in patients with CUPs. The purpose of this trial is to determine whether or not a strategy based on molecular analysis is effective in improving the progression free survival rates of patients with CUPs. The CancerTYPE ID is performed on formalin-fixed paraffin-embedded (FFPE) tumour specimens with at least 300 tumour cells (excluding decalcified bone samples). The analytical report indicated the location of the primary tumour with the highest probability rate and the primary sites that can be excluded.

More recently, a classifier of cancer types based on the microarray DNA methylation signature (EPICUP^®^, Bellvitge Biomedical Research Institute, Barcelona, Spain) has been proposed [74].

The assay showed 99.6% specificity and 97.7% sensitivity in a validation set of 7691 tumours. DNA methylation profiling predicted a primary cancer of origin in 188 (87%) of 216 patients with cancer with an unknown primary. Moreover, patients with an EPICUP diagnosis who received a tumour type-specific therapy showed improved overall survival compared with patients who received empiric therapy (HR, 3.24, *p* = 0.0051, 95% CI 1.42–7.38; log-rank *p* = 0.0029). The most frequent primary sites are, in descending order, pulmonary, bile ducts, upper aerodigestive tract, breast, colon, liver and pancreas [74,75].

Currently, another strategy is being implemented. The identification of “druggable” genomic mutations could be expected to propose an adapted treatment, not to the primary site but targeted to these alterations. A number of agents broadly targeting pathways critical to some cancers (i.e., EGFR or ALK inhibitors) have been incorporated into the standard therapy for various solid tumours. It is likely that some patients in the heterogeneous CUPs group would also benefit from these targeted agents [76].

A next-generation sequencing analysis (FoundationOne^®^, Foundation Medicine, Cambridge, MA, USA) of 237 genes showed that 96% of CUPs harboured at least one genetic alteration, with an average of 4 per tumour [2]. Of these, about 85% had an alteration that could be used to guide the treatment. For example, an alteration in the tyrosine kinase receptor/RAS pathway was observed in 72% of CUPs with an adenocarcinoma morphology. Nevertheless, a prospective study is needed to determine the clinical value of this strategy.

It should be noted, however, that the search for therapeutic targets by the pathologist should not replace the search for a primary origin, especially since the effectiveness of a targeted treatment for a mutation may vary according to the location of the cancer. In addition to diagnosis, the pathologist has an important role to play in optimal management of the tissue material, most often of biopsy samples [77]. One of the main causes of technical failure (15% to 25%) of molecular analyses is the low number of tumour cells in biopsies.

The extended diagnostic requirement of increasingly limited material provided by minimally invasive biopsy techniques and the cost-effectiveness of the DNA-based assays are major challenges for pathology departments. There is a need for an efficient diagnostic screening algorithm method of molecular alterations using IHC. IHC may provide an efficient screening tool for “druggable” genomic alterations by taking advantage of a growing list of available mutation-specific antibodies (i.e., *ALK* rearrangements, D5F3 and 5A4 clones; *ROS1* rearrangements, clone D4D6; *BRAF* V600E mutation, clone VE1; *EGFR* exon 19 E746_A750del, 6B6 clone; *EGFR* exon 21 L858R mutation, 43B2 clone) [78,79]. Recently, PD-1 and PD-L1, detected by IHC, have been identified as immune therapy biomarkers in various solid malignancies including CUPs, which may open up an unexplored avenue for treatment with anti-PD-1/PD-L1 antibodies in these patients [80,81].

In summary, the molecular approach enables the identification of the primary origin of a carcinoma when it is undifferentiated or does not express any specific markers of the primary origin. High throughput sequencing allows the detection of genetic “druggable” alterations to guide cancer therapy. Nevertheless, these new molecular approaches are expensive and the selected treatment must improve the outcome of patients, which remains to be demonstrated. Finally, the tissue material used for diagnosis and phenotyping should be used sparingly if a molecular analysis is expected.

## 7. Selected IHC Pitfalls

Aberrant or unexpected antigen expression should be considered as a source of a diagnostic pitfall in the evaluation of undifferentiated tumours [4]. In general, carcinomas express CK, whereas mesenchymal tumours express vimentin. However, there are carcinomas that show loss of CK expression, carcinomas that frequently co-express vimentin and carcinomas that rarely express both CK and vimentin. In contrast, mesenchymal tumours and hematopoietic neoplasms may express epithelial markers. A pitfall to keep in mind is that poorly fixed specimens may have an unpredictable pattern of staining for CK [4,9,82].

Non-epithelial tumours with a glandular epithelial component are extremely rare tumours, almost always biphasic, which likely distinguishes them from carcinomas. However, the partial and narrow nature of biopsy samples may sometimes be confined to the epithelial component and may mislead the diagnosis [82].

These biphasic tumours can be classified as “connective” and “non-connective.” The most common biphasic connective tumours are synovial sarcoma, dedifferentiated liposarcoma, mixed tumours/myoepithelioma of soft tissue, malignant peripheral nerve sheath tumour (or MPNST with glandular heterologous differentiation) and hamartomatous ectopic thymoma (Table 6). Non-connective tumours with a biphasic and/or mixed pattern are mainly represented by biphasic mesotheliomas, germ cell tumours and malignant mixed Mullerian tumours (carcinosarcomas and adenosarcoma with stroma overgrowth).

Synovial sarcoma represents 5% to 10% of all soft tissue sarcomas [84]. It is defined as a soft tissue tumour with a variable degree of epithelial differentiation, including gland formation. It is characterized by a specific translocation t(X;18)(p11;q11) (1). The biphasic form is quite common (30% to 50% of cases). This tumour occurs mainly between 15 and 40 years of age, without age predominance, by a deep mass, especially in the lower (85% to 95% of cases) or upper limbs (65% to 75%) but can occur in various anatomical sites [84]. Biphasic synovial sarcoma is usually recognized easily by the coexistence of a spindle cell component and a pseudo-glandular epithelial component. Vascularization is of a hemangiopericytic type, which should lead to the hypothesis of a synovial sarcoma. Epithelial cells are diffusely positive for AE1/AE3 pan-keratin and EMA. Conversely, spindle cells show almost constant positive reactivity with EMA, although focal, whereas only 70–80% of scattered cells are stained with AE1/AE3. A focal positivity of the spindle cells with S100 is found in 40% of cases. On the other hand, CD34 is almost always negative in both components and a positive CD34 rules out the diagnosis of synovial sarcoma [83]. More than 95% of synovial sarcomas are characterized by a recurrent, tumour-specific translocation t(X;18)(p11;q11), that can be detected by FISH in looking for the rearrangement in the *SS18* (*SYT*) gene [83].

Dedifferentiated liposarcoma is a high-grade non-lipogenic sarcoma that arises in a background of a pre-existing well-differentiated liposarcoma, harbouring a genomic amplification in the 12q13–15 region constituting the *MDM2*, *CDK4* and *HMGA2* genes [86]. IHC shows positive results for MDM2, CDK4 and HMGA2 [85,86,87]. The diagnosis is confirmed by the detection of *MDM2* and *CDK4* gene amplification. It occurs preferentially after 40 years in a retroperitoneal situation or in the deep soft tissues of the extremities. In exceptional cases, the dedifferentiated component can have variable trans-differentiation and may sometimes contain a glandular epithelial component [85,86,87].

The mixed tumour/myoepithelioma of soft tissue is a tumour with intermediate malignancy, occurring at any age, mainly located in the limbs and in 50% of cases in subcutaneous tissues [89]. The morphological aspect is identical to the mixed tumours of the salivary glands. The presence of a ductal epithelial component in the form of tubes of various sizes (the term “mixed tumour” is then used) can cause misdiagnosis on microbiopsies and raises the question of differential diagnosis of an adenocarcinoma, a biphasic synovial sarcoma or a MPNST with a “glandular” heterologous component [89]. IHC shows a mostly diffuse positivity to AE1/AE3, EMA and S100. These tumours sometimes express smooth muscle actin and p63 and in 50% of the cases GFAP. Between 10% and 40% of malignant forms show a loss in INI-1 expression unlike synovial sarcomas that show a decrease in INI-1 expression but with no loss of INI-1 expression [88]. About half of soft tissue myoepitheliomas show specific translocations involving the *EWSR1* gene with variable partners such as the *POU5F1*, *PBXI* and *ZNF444* genes [94]. These translocations can be detected by FISH by identifying a rearrangement in the *EWSR1* gene [94].

MPNSTs with glandular heterologous differentiation occurs, in 50% of the cases, in a context of type 1 neurofibromatosis [91]. Even if a heterologous component is observed in 10–20% of MPNSTs, glandular differentiation is rare. These glands have an enteric phenotype: CK20+ “goblet-cells” with neuroendocrine differentiation. The spindle cell component is generally negative for epithelial markers (AE1/AE3 and EMA). In addition, the spindle cell component presents a heterogeneous positivity in about 50% of cases with S100, which is quite similar to that observed in synovial sarcoma. The search for the *SS18* gene rearrangement gives a reliable diagnosis: the presence of a SS18 gene rearrangement in synovial sarcomas and the absence in MPNSTs [90,91].

Hamartomatous ectopic thymoma is a benign tumour, probably originating from the branchial pouch endoderm rather than from thymic remnants and is currently considered as a form of mixed tumour. It typically affects the middle-aged male adult (40 years of age) generally in the form of a 5 cm mass on average, occurring either superficially or deep in the area of the sternoclavicular joint. The histological appearance consists of solid islands of squamous epithelium that blend with spindled cells. Cysts lined by a squamous epithelium, small glands and fat also occur in variable amounts. Both the spindled and epithelial regions of the tumour express cytokeratins (AE1/AE3, CK5/6, CK14) and smooth muscle actin but neither Desmin nor S100 protein, while the fibroblast-like spindle cells are positive for CD34 [95].

The epithelial component of the biphasic mesothelioma retains positivity for AE1/AE3, CK5/6, calretinin and nuclear WT1, while sarcomatoid areas may lose these markers [31].

Germ cell tumours mainly concern testicular tumours of young men between 20 and 40 years of age but they are also reported at extra-gonadic sites such as the retroperitoneum and mediastinum (migration sites of primary germ cells) [92]. A change in serum markers (AFP and β-HCG) can be detected. Morphologically, this tumour is composed of a variable mixture of seminomatous and non-seminomatous elements (embryonic carcinoma, choriocarcinoma, teratoma). Occasionally, malignant transformation of teratomatous elements can be observed, especially in sarcomatoid components, in particular rhabdomyosarcoma. Most germ cell tumour components are positive for SALL-4 and pan-keratin, whereas seminomatous elements are positive for KIT (CD117) and OCT-4 [92].

Malignant mixed Mullerian tumours (“carcinosarcoma”) are uterine tumours of the nulliparous and postmenopausal women (median age 65 years), sometimes occurring in the ovaries, fallopian tubes, cervix or peritoneum [93]. Morphologically, these tumours present a variable proportion of carcinomatous components (serous or endometrioid carcinoma to be investigated by careful sampling) and of malignant connective components (proliferation of spindle, pleomorphic and undifferentiated cells occasionally with heterologous elements such as rhabdomyo-, léiomyo-, chondro or osteosarcoma). The carcinomatous component is positive with pan-keratin (AE1/AE3), PAX8 and WT1 (serous carcinoma). Conversely, the sarcomatoid component presents variable expression of cytokeratins but can express specific differentiation markers (Desmin, Myogenin, Caldesmon, or S100 protein, depending on the proportion of heterologous elements) [93].

Finally, when dealing with limited narrow biopsies showing a malignant neoplasm with an adenocarcinoma morphology, a likely biphasic soft tissue tumour should always be kept in mind. It is necessary to obtain a minimum of clinical information, to carry out rigorous morphological analysis of each component (including assessment of the monomorphic or pleomorphic aspect of the spindle component) and to use a relevant antibody panel to guide any molecular analysis.

## 8. Conclusions

In conclusion, in the era of expanding knowledge into tumour genomics and into the immune microenvironment as well as into associated targeted therapy, the role of the pathologist is expanding [82]. A systematic approach, beginning with histomorphological evaluation, the algorithmic application of a small and effective panel of immunomarkers and the precise selection of molecular or other ancillary tests, is required for the efficient management of CUPs. The organ- or tumour-specific immunomarkers, including some newer generations of immunomarkers, help to obtain a specific diagnosis. Only by exercising a systematic approach to working up CUPs can an accurate specific diagnosis be reached. A systematic approach is also required to preserve tissue for potential molecular or other ancillary testing for most cases in the routine practice.

## Figures and Tables

**Table 1:** Main primary origins of carcinomas of unknown primary site (CUPs) based on staining for CK7 and CK20 [4,8].

CK7+/CK20−	CK7+/CK20+	CK7−/CK20+	CK7−/CK20−
Breast carcinoma Lung adenocarcinoma Endometrial adenocarcinoma Endocervical adenocarcinoma Ovarian (serous) carcinoma Cholangiocarcinoma Small cell lung carcinoma Mesothelioma Thyroid carcinoma Salivary gland tumours Kidney (papillary) Urothelial carcinoma (subset) Pancreatic adenocarcinoma Gastric adenocarcinoma	Urothelial carcinoma Pancreatic adenocarcinoma Ovarian mucinous carcinoma Bladder adenocarcinoma Gastric adenocarcinoma Cholangiocarcinoma	Colorectal adenocarcinoma Merkel cell carcinoma Gastric adenocarcinoma	Prostate adenocarcinoma Renal (clear cells) Hepatocellular carcinoma Adrenocortical carcinoma Non-seminoma germ cell tumours Mesothelioma Small cell lung carcinoma Gastric adenocarcinoma

**Table 2:** Immunohistochemistry (IHC) tumour staining patterns in the differential diagnosis of CUPs expressing CK7+/CK20− [7].

Primary Site of Origin	Immunostaining Profile
Breast [8,14,15,16,17]	ER+/PgR+, GATA3+, GCDFP15−/+, MGB+/−, TFF1−
Ovary (serous) [17,18,19,20,21]	PAX8+, ER+, WT1+, TTF1−, TFF3−, GATA3−
Ovary (clear cell) [17,18,19,20,21]	pVHL+, HNF-1β+, Napsin A+, AFP−, WT1−, ER−, GPC3−
Endometrium [17,18,19,20,21]	ER+, PAX8+, Vimentin+
Uterine cervix [17,18,19,20,21]	p16+, HPV+, CEA+, PR−, PAX2−, PAX8+/−
Lung [22,23,24]	TTF1+, Napsin A+, GATA3−
Thyroid (papillary/follicular) [23,24,25]	TTF1+, Thyroglobulin+, PAX8+
Thyroid (medullary) [23,24,25]	TTF1+, Calcitonin+, CEA+
Stomach [26,27,28,29]	CEA+, CDX2−/+, MUC1−/+, MUC5AC−/+, CDH17+/−, TTF1−
Oesophagus [26,27,28,29]	CDX2+/−, CEA+, CDH17+, MUC1−/+, MUC5AC−/+, SATB2−
Pancreas [26,27,28,29]	DPC4−/+, CK17+/−, pVHL−, Maspin+, S100P+, MUC5AC+
Urinary bladder [17,18,19,20,21]	GATA3+, p63+, CK5/6+, p40+, S100P+, CK903+, UPII+/−
Thymus [19,20,21]	CD5+/−, p63+/−, PAX8+/−, CD117+/−, Glut1+/−
Salivary (ductal) [16,17,30]	GATA3+, AR+, GCDFP-15+
Mesothelioma [30,31,32,33,34]	Calretinin+, WT1+, CK5/6+, TTF1−, CEA−, BerP4−

Abbreviations: AR, androgen receptor; calretinin; AFP, α-fetoprotein; CD5, cluster of differentiation 5; CDH17, cadherin-17; CDX2, caudal type homeobox 2; CEA, carcinoembryonic antigen; CK, cytokeratin; D2-40, podoplanin; DPC4, SMAD family member 4; ER, oestrogen receptor; GATA3, GATA binding protein 3; GCDFP-15, gross cystic disease fluid protein 15; HNF-1b, hepatocyte nuclear factor 1b; HPV, human papillomavirus; MGB, mammaglobin; MUC, mucin; PAX, paired box gene; CEA, carcinoembryonic antigen; PgR, progesterone receptor; pVHL, von Hippel-Lindau tumour suppressor; S100P, placental S100; TFF, trefoil factor; TFF3, trefoil factor 3; TM, thrombomodulin; TTF1, thyroid transcription factor 1; UPII, uroplakin II; WT1, Wilms tumour 1.

**Table 3:** Malignant peritoneal mesothelioma (MPM) versus papillary serous peritoneal carcinomas (PSPCs) and non-gynaecological adenocarcinomas (ADCs) [31,32].

**Mesothelial Markers**	
Calretinin	Useful. Positive in 85–100% of MPMs. Positivity in 0–38% of PSPCs prevents its use as a single differential marker.
D2-40	Potentially useful. Positive in 93–96% of MPMs but also focal positivity in 13–65% of PSPCs; additional data are needed.
CK5/6	Limited use. Positive in 53–100% of MPMs but also focal positivity in 22–35% of PSPCs.
WT1	Not useful. Positive in 43–93% of MPMs and positive in 89–93% of PSPCs.
**PSPCs Markers**	
MOC-31	Very useful. Positive in 98% of PSPCs and 5% of MPMs.
PAX8	Very useful. Positive in most Mullerian carcinomas and negative in MPMs.
BG8	Very useful. Positive in 73% of PSPCs and 3–9% of MPMs.
BerEP4	Useful. Positive in 83–100% of PSPCs and in 9–13% of MPMs.
B72.3	Limited use. Positive in 65–100% of PSPCs and focal expression in 0–3% of MPMs.
CEA	Not useful. Positive in only 0–45% of PSPCs and negative in MPMs but sensitivity too low compared to other markers.
Oestrogen receptor	Useful. Positive in 60–93% of PSPCs and 0–8% of MPMs.
Progesterone receptor	Limited use. Lower sensitivity than oestrogen receptors in PSPCs, negative in MPMs. Can be useful when positive.
**Mesothelioma vs. Non-Gynaecological Adenocarcinoma (Biliary, Pancreas, Stomach, Colon)**	
Calretinin	Very useful. Positive in 85–100% of MPMs but also positive in 10% of pancreatic ADCs, limited value as single marker.
WT1	Very useful. Positive in 43–93% of MPMs, 3% of gastric ADCs and 0% of pancreatic ADCs.
D2-40	Potentially useful. Positive in 93–96% of MPMs, negative in gastric and pancreatic ADCs (limited data).
CK5/6	Not useful. Positive in 53–100% of MPMs and 38% of pancreatic ADCs.
MOC-31	Very useful. Positive in 5% of MPMs and 87% of ADCs.
BG8	Very useful. Positive in 3–9% of MPMs and 89% of ADCs.
CEA	Very useful. Negative in MPMs and positive in 81% of ADCs.
B72.3	Very useful. Positive in 0–3% of MPMs, 84% of pancreatic ADCs, 89% of biliary ADCs, 98% colon ADCs.
BerEP4	Useful. Positive in 9–13% of MPMs et >98% pancreatic and gastric ADCs.
CDX2	Useful. Positive in 90–100% of colon ADCs, 80% small intestine ADCs, 70% of gastric ADCs and negative in MPMs.

**Table 4:** IHC tumour staining patterns in the differential diagnosis of CUPs expressing CK7+/CK20+ [1,7].

Primary Origin Site	Immunostaining Profile
Lung (mucinous) [49]	TTF1−/+, CK7−/+, CDX2−/+
Pancreas [26,27,50,51]	Maspin A+, S100P+, IMP-3+, pVHL−, SMAD4−/+, MUC5AC+, CDX2−/+
Stomach [26,27,50,51]	CEA+, CDX2−/+, MUC1−/+, MUC5AC−/+, CDH17+/−, TTF1−
Oesophagus [26,27,50,51]	CEA+, MUC5AC+/−, CDH17+, MUC1−/+, CDX2−/+
Ovary (mucinous) [7,52]	DPC4+, CA-12.5+, CDX2+/−
Urinary bladder [17,18,19,20,21]	GATA3+, p63+, p40+, CK5/6+, CK20+/−, S100P+, CK903+, UPIII+/−
Small intestine [39]	CDX2+, CDH17+, Villin+/−, MUC5AC+/−
NUT midline carcinoma [53,54,55]	CK7+/−, CK20+/−, p40+/−, NUT

**Table 5:** IHC tumour staining patterns in the differential diagnosis of CUPs expressing CK7−/CK20− [1,7].

Primary Site	IHC Profile
Prostate [58,59,60,61,62]	PSA+, NKX3.1+/−, PSAP+, P504S+, ERG+/−
Colon (medullary) [29,35,36]	SATB2+, CDH17+, TFF3+/−, Calretinin+/−, CDX2−/+
Renal [21,63]	CD10+, PAX8+, Vimentin+, pVHL+, RCCMa+, Inhibin−, TTF1−, CEA−
Liver [64]	HepPar1+, CD10+, pCEA+, mCEA−, AFP+, Glypican-3+, Arginase-1+, CK19−
Adrenal (cortical) [4,7,10]	Melan A+, Calretinin+, Inhibin A+, Synaptophysin+, Chromograni−, CEA−
Germ cell tumours [4,7,10]	CD117+, OCT4+, CD30+, Glypican-3+, PLAP+, SALL4+, NANOG+

**Table 6:** Tumour-specific markers useful for the diagnosis of various biphasic connective tumours.

Type of Tumour	IHC Markers
Synovial sarcoma [83,84]	AE1/AE3+/−, EMA+/− (epithelial cells), S100+ (spindle cells), CD34+
Dedifferentiated liposarcoma [85,86,87]	MDM2+, CDK4+, HMGA2+
Mixed tumour/myoepithelioma of soft tissue [88,89]	AE1/AE3+, EMA+, S100+, SMA+/−, p63+/−, GFAP+/−, INI-1−
MPNST [90,91]	S100+, AE1/AE3−, EMA− (spindle cells)
Hamartomatous ectopic thymoma [92]	AE1/AE3+, CK5/6+, CK14+, SMA+, CD34+ (spindle cells), Desmin−, S100−
Biphasic mesothelioma(epithelial component) [31,32]	AE1/AE3+, CK5/6+, Calretinin+, WT1+
Germ cell tumours [92]	AE1/AE3+, SALL-4+, CD117+, OCT-4+
Malignant mixed Mullerian tumours (“carcinosarcoma”) [93]	AE1/AE3+, PAX8+, WT1+Sarcomatoid components: Desmin+, Myogenin, Caldesmon+, S100+
